# Lack of Infection with XMRV or Other MLV-Related Viruses in Blood, Post-Mortem Brains and Paternal Gametes of Autistic Individuals

**DOI:** 10.1371/journal.pone.0016609

**Published:** 2011-02-23

**Authors:** Carla Lintas, Francesco Guidi, Barbara Manzi, Antonio Mancini, Paolo Curatolo, Antonio M. Persico

**Affiliations:** 1 Laboratory of Molecular Psychiatry and Neurogenetics, University Campus Bio-Medico, Rome, Italy; 2 Laboratory of Molecular Psychiatry and Psychiatric Genetics, Department of Experimental Neurosciences, I.R.C.C.S. “Fondazione Santa Lucia”, Rome, Italy; 3 Institute of Hematology, Catholic University of the Sacred Heart, Rome, Italy; 4 Department of Child Neuropsychiatry, University “Tor Vergata”, Rome, Italy; 5 Department of Internal Medicine, Catholic University of the Sacred Heart, Rome Italy; University of Minnesota, United States of America

## Abstract

**Background:**

Autistic spectrum disorder (ASD) is characterized by impaired language, communication and social skills, as well as by repetitive and stereotypic patterns of behavior. Many autistic subjects display a dysregulation of the immune system which is compatible with an unresolved viral infection with prenatal onset, potentially due to vertical viral transmission. Recently, the xenotropic murine leukemia virus-related virus (XMRV) has been implicated in chronic fatigue syndrome (CFS) and in prostate cancer by several, though not all studies.

**Methodology/Principal Findings:**

We assessed whether XMRV or other murine leukemia virus (MLV)-related viruses are involved in autistic disorder. Using nested PCR targeted to *gag* genomic sequences, we screened DNA samples from: (i) peripheral blood of 102 ASD patients and 97 controls, (ii) post-mortem brain samples of 20 ASD patients and 17 sex- and age-matched controls, (iii) semen samples of 11 fathers of ASD children, 25 infertile individuals and 7 fertile controls. No XMRV *gag* DNA sequences were detected, whereas peripheral blood samples of 3/97 (3.1%) controls were positive for MLV.

**Conclusions|Significance:**

No MLV-related virus was detected in blood, brain, and semen samples of ASD patients or fathers. Hence infection with XMRV or other MLV-related viruses is unlikely to contribute to autism pathogenesis.

## Introduction

Autism Spectrum Disorder (ASD) is a complex neurodevelopmental disorder, characterized by different levels of impairment in social interaction and communication, as well as by stereotypies and rigid patterns of behaviour [1]. Disease onset occurs prior to 3 years of age and its incidence is currently estimated at 1/150 live births [2]–[3]. ASD is the most heritable neuropsychiatric disorder, yet very few cases can be solely explained on the basis of *de novo* genetic mutations or cytogenetic abnormalities [4]. Vertical viral transmission represents a non-genetic mechanism compatible with high parent-to-offspring transmission and with low rates of disease-specific genetic abnormalities [5]. Clinically, many ASD patients display a dysregulation of the immune system, potentially suggestive of a prenatal-onset, unresolved viral infection [6]–[7]. Vertically transmitted viruses should be found more frequently in the affected tissues of autistic individuals compared to controls: based on this hypothesis we initially assessed the prevalence of several neurotropic viruses in post-mortem brains of autistic patients and controls, finding a significant association between ASD and polyomavirus infection [8]. In the present study, we focus our attention on xenotropic murine leukemia virus-related virus (XMRV) and other xenotropic murine leukemia (MLV)-related viruses. These retroviruses indeed represent good candidates for vertical viral transmission in autism, because of their ability to integrate into the parental host genome and thus undergo parent-to-child transmission. Furthermore, XMRV infection is currently a source of serious concern in the USA for its possible link with chronic fatigue syndrome (CFS) [9].

Using nested PCR, XMRV and MLV *gag* genomic sequences were sought in the following biological samples: (a) peripheral blood mononuclear cells (PBMC) belonging to 102 ASD patients and 97 controls, (b) post-mortem brains of 20 ASD patients and 17 sex- and age-matched controls, and (c) semen samples belonging to 11 fathers of ASD children, 25 infertile individuals and 7 fertile controls. Our results do not support the frequent involvement of XMRV or MLV-related viruses in autism pathogenesis.

## Methods

### Patients and samples

All subjects, except for post-mortem brain donors, were recruited in Italy and are ethnically Italian. The demographic characteristics of these samples are summarized in Table 1. Briefly, (a) PBMC were obtained drawing blood from ASD patients diagnosed for any ASD (either Autistic Disorder, Asperger Disorder, or Pervasive Developmental Disorder Not Otherwise Specified) according to DSM-IV criteria [1], and clinically assessed as described [10]. Controls were drawn as prescribed by family practitioner for a broad range of physical complaints unrelated to psychiatric disorders and among nursing and medical students at University Campus Biomedico (Rome, Italy), as described [10]; (b) frozen *post-mortem* brain tissues dissected from the superior temporal gyrus (Brodmann Areas 41/42 or 22) were obtained through the Autism Tissue Program from the NICHD Brain & Tissue Bank (Baltimore, MD) and the Harvard Brain Tissue Resource Center (Belmont, MA). These tissue samples largely overlap with those employed in our previous studies [10], as this neocortical region hosts well-documented structural and functional abnormalities in autism [11]; (c) semen samples were provided by outpatients who underwent andrological evaluation for infertility at the Division of Endocrinology of Catholic University of the Sacred Heart (U.C.S.C., Rome, Italy) upon vibratory stimulation using Ferticare Clinic (Multicept, Frederiksberg, Denmark), according to the ethical guidelines approved by the Institutional Review Boards (IRB) of Catholic University of the Sacred Heart and University Campus Bio-Medico (U.C.B.M.). Within 1 hour from collection, semen specimens were separated into seminal fluid and three cellular fractions (mobile sperm cells, sperm cells with hypomotility, and non-mobile cells including immobile spermatozoa, immature forms, leukocytes and epithelial cells) by centrifugation at 300 g for 30 minutes using Isolite® (IrvineScientific, Santa Ana, CA, USA); aliquots were stored at −80°C until DNA extraction. The consent forms signed by all individuals involved in blood and semen collection, including parents for their children, were approved by the Institutional Review Board of University Campus Bio-Medico (U.C.B.M.).

**Table 1:** Demographic characteristics of the samples used in this study.

Sample type (N)		Status (N)	Mean age ± SD (year)	Sex
		ASD^1^ (N = 20)	15.4±9.5	M∶F = 15∶5
Post-mortem brains (N = 37)				
		Controls (N = 17)	17.2±8.5	M∶F = 12∶5
		ASD (N = 102)	10.2±5.4	M∶F = 83∶19
PBMCs^2^ (N = 199)				
		Controls (N = 97)	50.0±15.7	M∶F = 45∶52
	mobile spermatozoa (N = 8)			
	hypomobile spermatozoa (N = 8)	ASD fathers (N = 9	43.4±9.5	-
	non-mobile cells (N = 9)			
	mobile spermatozoa (N = 19)			
Semen (N = 110)	hypomobile spermatozoa (N = 20)	Infertile (N = 25)	35.1±6.1	-
	non-mobile cells (N = 25)			
	mobile spermatozoa (N = 7)			
	hypomobile spermatozoa (N = 7)	Fertile (N = 7)	33.8±3.5	-
	non-mobile cells (N = 7)			

1 ASD, Autistic Spectrum Disorder.

2 PBMCs, Peripheral Blood Mononuclear Cell.

### Nested PCR and sequencing

DNA was recovered by phenol/chloroform extraction and ethanol precipitation, following cell digestion with proteinase K at 55°C overnight. XMRV *gag* nested PCR was performed as previously described [12] with the following modifications: approximately 80 ng of genomic DNA in 25 µl final PCR reaction volume were used as a template for the first round PCR; 40 cycles were done for each round of amplification. In our hands, nested PCR sensitivity was at 10 viral copies, as in previous reports [13]. Each PCR experiment included equal numbers of patients and controls, as well as negative controls for the first and second round PCRs; in order to minimize the risk of contaminations, positive controls were PCR-amplified separately and run on the same agarose for band size determination, as in our previous studies [8]. Appropriately-sized PCR products (413 bp) were sequenced, using a CEQ8000 DNA sequencer (Beckman-Coulter, Fullerton, CA). In order to exclude contaminations with mouse genomic DNA, MLV positive samples were also assessed by a nested PCR targeting mouse histone deacetylase 5 (Hdac5) and displaying the same sensitivity as the nested PCR used to detect MLV.

## Results

**Figure 1:**
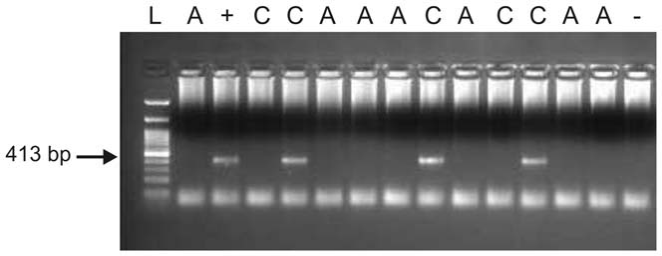
XMRV *gag* nested PCR on PBMC DNA, showing positive samples in lane 5, 9 and 12. A = autistic, C = control, L = ladder (100 bp GeneDirex), + = positive control, − = negative control.

No MLV-related virus *gag* sequences were detected in 96 blood samples and 20 post-mortem brains of ASD patients, as well as in 25 semen fractions belonging to 9 fathers of ASD children (Table 1). Similarly, 17 control brains, and 85 semen fractions from 7 fertile and 25 infertile controls (Table 1) were negative for MLV-related *gag* sequences. Three out of 97 (3.1%) peripheral blood samples from unaffected controls were positive (Figure 1). The difference between ASD blood samples and unaffected controls does not reach statistical significance (Fisher's exact P-value = 0.25, n.s.). DNA sequencing and BLAST analysis unveiled in 3 control blood samples viral *gag* gene sequences displaying 100% alignment with the mouse endogenous retrovirus MLV on chromosome 8 (GenBank Acc number: AC163617 nt 85467–85880). No mouse genomic contamination was detected in these three positive MLV control samples by nested PCR targeting the mouse histone deacetylase 5 (Hdac5) gene.

## Discussion

Our results show that infection with XMRV or other MLV-related viruses, assessed both in the central nervous system and in blood, is not associated with ASD nor is likely implicated in vertical viral transmission through parental gametes. We thus replicate and largely extend a recent study reporting no association between XMRV infection and autistic disorder [14].

A search for viruses as primary etiological agents in autism is well justified. Congenital infection with rubella or cytomegalovirus (CMV) represents one of the best-documented environmental factors significantly associated with ASD (for review see [15], [16]). The largest longitudinal study involving several hundred children prenatally exposed to rubella virus estimates at 7.4% the rate of autism in this group, much higher than ASD prevalence rates in the general population; risk appears especially high if rubella infection occurs during the first 8 weeks postconception [17]–[20]. Evidence linking prenatal CMV infection to autism is more circumstantial, but several case reports have been published [21]–[28]. Risk estimates are essentially based on a small cohort of 7 prenatally CMV-infected children, who displayed autistic features in 2 cases (2/7 = 28.6%) [25].

XMRV represents an interesting candidate to potentially play a role in autism pathogenesis. It was initially identified by PCR in approximately 10% of prostate cancer patients [12]. It is phylogenetically related to MLV-related viruses and displays about 90% sequence identity with MLV [12]. Recently, XMRV infection has been strongly associated with CFS [9]. Attempts to replicate these initial results in European and North-American cohorts of prostate cancer and CFS patients have yielded conflicting results. In general, the association between XMRV infection and human disease appears stronger in the USA compared to Europe (Table 2). However, also four US studies are completely negative [13], [14], [29], [30], accounting for about two thirds of the total patient sample recruited in North America (Table 2). The discrepancy between European and North American studies could therefore reflect differences in PCR-based assay sensitivity rather than real geographical differences in the prevalence of infection by XMRV or other MLV-related viruses. In this respect, it will be important to establish and validate universal assays, as recently proposed by the National Institutes of Health.

**Table 2:** Studies on XMRV and/or MLV-related virus in several pathologies, by country of origin of the sample.

Ref.	Country	Pathology	Tissue	Patients	Controls	Virus
[12]	USA	Prostate cancer	Prostate tissue	9/86 (10%)	-	XMRV
[9]	USA	CFS^1^	PBMC^2^	68/101(67%)	8/218(3.7%)	XMRV
[32]	USA	Prostate cancer	Prostate tissue	14/233 (6%)	2/101 (2%)	XMRV
[33]	USA	CFS	PBMC	32/37(86.5%)	3/44 (6.8%)	MLV-related
[13]	USA	CFS	PBMC	0/50 (0%)	0/97 (0%)	-
[31]	USA	Prostate cancer	Prostate tissue	32/144 (22%)	-	XMRV
[29]	USA	CFS, HIV, RA^3^	PBMC	0/293 (0%)	-	-
[30]	USA	Prostate cancer	Prostate tissue	0/800 (0%)	-	-
[14]	USA	ASD^4^	PBMC	0/134 (0%)	0/204(0%)	-
	**TOTAL USA^7^**			**155/1878**	**13/664**	
				**(8.2%)**	**(2%)**	
[14]	Italy	ASD	PBMC	0/96 (0%)	-	-
[34]	Netherlands	CFS	PBMC	0/32 (0%)	0/43 (0%)	-
[35]	UK	CFS	PBMC	0/186 (0%)	-	-
[36]	Netherlands	Prostate cancer	Prostate tissue	3/74 (4%)	-	XMRV
[37]	UK	CFS	PBMC	0/108 (0%)	-	-
[38]	Germany	Prostate cancer	Prostate tissue	1/105 (1%)	1/70 (1.4%)	XMRV
[39]	Germany	Prostate cancer	Prostate tissue	0/589 (0%)	-	-
[40]	China	CFS	PBMC, plasma	0/65 (0%)	0/85 (0%)	-
[41]	Netherlands	HIV	Seminal plasma	0/54 (0%)	-	-
[42]	France	ID^5^ & others	PBMC & others	0/62 (0%)	0/99 (0%)	-
[43]	Germany	RTI^6^	Resp.secretions	20/267(7.4%)	2/62 (3%)	XMRV
[44]	UK	HIV and HCV	PMBC, plasma	0/232 (0%)	-	-
	**TOTAL REST OF THE WORLD^7^**			**24/1870**	**3/359**	
				**(1.3%)**	**(0.8%)**	

Studies were based on nested PCR or real time PCR (genomic or RT-PCR).

1 CFS, Chronic Fatigue Syndrome,

2 PBMC, Peripheral Blood Mononuclear Cells,

3 RA, Rheumatoid Arthritis,

4 ASD, Autistic Spectrum Disorder,

5 ID, Infiammatory Diseases,

6 RTI, Respiratory Tract Infections.

7 Patients vs controls - USA: χ^2^ = 30.49, df = 1, P = 3.35×10^−8^; Rest of the world: χ^2^ = 0.504, df = 1, P = 0.477, n.s.

USA vs Rest of the World - patients: χ^2^ = 98.57, df = 1, P = 3.13×10^−23^; controls: χ^2^ = 1.900, df = 1, P = 0.170, n.s.

Our results, combined with those reported by Sutherfield et al [14], render XMRV contributions to autism highly unlikely. Nonetheless we cannot exclude that MLV-related viruses may play a role in rare cases.
